# Improving the physicochemical and antioxidative properties of fermented goat milk using carob molasses and some probiotic strains

**DOI:** 10.1007/s10068-023-01382-2

**Published:** 2023-08-25

**Authors:** Mahmoud Ibrahim El-Sayed, Esmat Aly, Amany Mohammed El-Deeb

**Affiliations:** Dairy Technology Research Department, Food Technology Research Institute, Agricultural Research Center, Giza, 12613 Egypt

**Keywords:** Carob molasses, Probiotic viability, Fermented goat milk, Antioxidant, Sensory properties

## Abstract

Fermented goat milk samples, processed by adding 0, 2, and 4% carob molasses, and *Bifidobacterium bifidum DSMZ* or *Lactobacillus helveticus CH5*, were evaluated for their physicochemical, antioxidative, and sensory properties along with probiotic viability during storage. pH and viscosity gradually decreased over the storage period whereas their values increased with the increased amount of carob molasses. *Bifidobacterium bifidum DSMZ* or *Lactobacillus helveticus CH5* kept higher viable numbers. *Lactobacillus helveticus CH5* strain recorded higher viability. Increasing the added amount of carob molasses increased the total phenolic content, which in turn is reflected in the increase in antioxidant activity. Adding carob molasses participated in the partial masking of the goaty flavor and had no significant effect on the product’s acceptability, which was strongly influenced by the progress of cold storage. Therefore, fermented goat milk described by its enhanced physicochemical and antioxidative properties could be obtained by adding carob molasses and probiotics.

**Supplementary Information:**

The online version contains supplementary material available at 10.1007/s10068-023-01382-2.

## Introduction

Nowadays, goat milk represents the third most-produced milk type worldwide after cow and buffalo milk. Goat milk products have received more attention and thus gained more popularity due to the recent development in food technology along with the accumulated knowledge about its nutritional attributes and health benefits. This could be explained by its smaller fat droplets, lower amounts of α-S1-casein, and greater micellar dispersion, contributing to its lower allergenicity and easier digestion, as compared to cow milk (Siriwardhana et al., 2021). Besides, goat milk represents a suitable carrier for probiotic delivery due to its dense nutritious composition, proper pH, and good buffering capacity (Jacquot et al., 2015). It can be used, at the industrial level, in manufacturing several products such as pasteurized or sterilized milk, fermented milk, and ice cream among others (Fazilah et al., 2018). However, the unpleasant goaty flavor represents its main defect which lowers its consumer perception and acceptability (Wang et al., 2019).

Given that milk products are well-known for not containing polyphenols substances, several plants’ polyphenols-rich extracts were employed as functional components in dairy products for providing health advantages and thereby increasing consumer interest (El-Sayed et al., 2019). In this context, carob pods are considered a prominent ingredient with many health-improving properties due to their high content of sugars and phytochemicals (Ibrahim et al., 2020). Carob pod’s water-soluble extract is a rich source of polyphenols, antioxidants, polysaccharides, and dietary fiber, which is translated to multiple physiological functions including antioxidant, hypoglycemic, antimicrobial, and anti-inflammatory effects (Rtibi et al., 2017). Carob molasses, another product prepared from carob pod, is described by its dark color, high sugar content, and health-promoting properties, e.g. antioxidant, and also a strong emulsifying ability (Tounsi et al., 2020). The various properties of carob molasses strengthen its importance as a natural additive to improve the quality attributes of goat milk products.

Like other dairy products, goat milk products represent an effective delivery system for probiotic bacteria which are well-known for their wide range of health-promoting activities that are driven through several mechanisms, including modulation of intestinal microflora, anti-cancer activity, and adhesion to colon cancer cells (Terpou et al., 2019). These health benefits of probiotics are strain-specific. Consequently, various bacterial strains of the same species may encourage completely different characteristics and have different impacts on the host (Mantzourani et al., 2019). Therefore, the microorganism strains need to be identified and tested in a case-by-case approach and evaluate their specific characteristics and their potential positive impacts on human health (Ruiz-Moyano et al., 2019).

Nowadays, several attempts are recently carried out to improve the physicochemical, texture and sensory attributes, and functionality of fermented goat milk products by using derivatives of food of plant origin and/or probiotic bacteria. Given the above-mentioned, this investigation was carried out to improve some selected physicochemical and antioxidative properties along with the sensory properties of fermented goat milk by adding carob molasses combined with some probiotic strains.

## Material and methods

### Reagents, chemicals, and cultures

1,1-diphenyl-2-picryl-hydroxyl (DPPH) and Folin–Ciocalteau reagents were obtained from Sigma–Aldrich (Munich, Germany). Ferric chloride, potassium ferricyanide, and gallic acid were obtained from Loba Chemie, Mumbai, India. All other solvents, chemicals, and culture media used were of analytical grade. *Lactobacillus helveticus CH5* was obtained from the Egyptian Microbial Culture Collection (EMCC) belonging to Cairo Microbial Resources Center (MIRCEN), Faculty of Agriculture, Ain Shams University, Egypt while *Bifidobacterium bifidum DSMZ* was kindly provided by Food Science and Human Nutrition Dept. (NUTBRO group), Faculty of Veterinary Sciences, University of Murcia, Spain.

### Preparation and characterization of carob molasses

Carob pods were purchased from a local market in Aswan city, Egypt, and were used in preparing the carob molasses according to the method of Dhaouadi et al. (2014). Briefly, the pods were washed with tap water to remove the dirt and dust and then air-dried. The seeds were manually separated from pods before milling to pass through a sieve (40 mesh). Carob powder was mixed with tap water at a ratio of 1:2 (w/v) and the extraction was allowed for 24 h at 4 °C, and then filtered through cheesecloth. The carob extract was concentrated under vacuum at 50 °C for 48 h using a lab vacuum oven (Model 3618, USA). The obtained molasses was packed in brown glass bottles and stored at 4 ± 1 °C until use.

Carob molasses contents of total soluble solids, total sugars, ash, and mineral have been estimated according to AOAC (2005). Its content of individual polyphenols has been identified according to Schieber et al. (2001) using HPLC Agilent 1260 series equipped with an Eclipse C18 column (4.6 mm × 250 mm i.d., 5 μm) which was maintained at 35 °C during analysis, auto-sampler, solvent degasser, ultraviolet (UV) detector set at 280 nm and quarter HP pump (series 1050). The mobile phase consisted of water (A) and 0.05% trifluoroacetic acid in acetonitrile (B) at a flow rate of 1 mL/min. The mobile phase was programmed consecutively in a linear gradient as follows: 0 min (82% A); 0–5 min (80% A); 5–8 min (60% A); 8–12 min (60% A); 12–15 min (85% A) and 15–16 min (82% A). The injection volume was 10 μl for each of the sample solutions. External standards were used in the identification and quantification of all individual polyphenols.

### Preparation of bio-fermented goat milk

Fresh goat milk, obtained from El-Serw Animal Production Research Station, Animal Production Research Institute, Agricultural Research Center, Egypt, has been used in preparing various samples of bio-fermented goat milk. Goat milk has been incorporated with 0, 2, and 4% carob molasses based on a previous preliminary sensory evaluation study to select the most acceptable supplemented fermented goat milk. The goat milk has been thermally treated at 80 °C for 5 min. After cooling to 40 °C, milk has been divided into six equal portions: three portions were inoculated with 3% of the activated *B. bifidum* DSMZ and 0, 2, and 4% carob molasses (they were coded as follows: CB, CB2, and CB4, respectively). The other three treatments were inoculated with 3% of the activated *L. helveticus* CH5 and 0, 2, and 4% carob molasses (they were coded as follows: CH, CH2, and CH4, respectively). Samples named CB and CH served as control samples in the current study. All samples have been incubated at 40 °C until pH falls to 4.6. The resultant fermented goat milk samples were kept in the refrigerator at 4 ± 1 °C. The next day, the sucrose solution (prepared by mixing 100 g sucrose with 100 mL water followed by heating it at 85 °C for 5 min and then cooling it) was incorporated at a level of 6% and blended well with the fermented milk product. The obtained sweetened bio-fermented goat milk beverages were divided and packed into 100 mL sterilized glass bottles and stored at 4 °C. All analyses were conducted in triplicate at 1, 7, and 14 days of cold storage.

### Physicochemical analyses of bio-fermented milk

The bio-fermented goat milk samples have been analyzed for total solids (%), protein (%), ash %), and fat (%) according to AOAC (2005). The pH and viscosity values of fermented milk samples were measured using a digital pH meter (Jenway, UK) and an oscillatory viscometer (Model NDJ-9S, U.S.A) using spindle 3 at speed of 60 rpm/10 °C, respectively.

### Viability of probiotic bacteria

Viable counts of *Bifidobacterium bifidum* DSMZ and *L. helveticus* CH5 have been determined using MRS agar (Merck, Germany) under anaerobic conditions at 37 °C for 48 h according to IDF (1988). The viable numbers of probiotic bacteria have been expressed as colony-forming unit per mL of the product (log CFU/mL).

### Phenolic extraction and antioxidant determination of carob molasses and fermented goat milk

For determining its total phenolic content (TPC) and antioxidant activity, carob molasses has been diluted 100 times using water. Five grams of fermented goat milk were well-mixed with 25 mL of methanol solution 75% (25 mL), then homogenized by Digital Homogenizer (PRO25D from Thomas Scientific, USA) and centrifuged (Sigma centrifuge 113, VWR International, Darmstadt, Germany) at 7000 rpm for 15 min/4 °C. The obtained supernatants have been filtered by Whatman No.1 and the obtained phenolic extracts were stored at 4 °C until analysis (Öztürk et al., 2018). The 100 × diluted extract of carob molasses and the obtained phenolic extract of each fermented milk have been used in determining the total phenolic content and antioxidant activity as follows:

Total phenolic contents (TPC) of carob molasses (diluted 100 times) and bio-fermented goat milk extracts were determined according to Abirami et al. (2014). Briefly, one and a half milliliters of Folin–Ciocalteau reagent (diluted 10 times) and 1.2 mL of Na_2_CO_3_ (7.5% w/v) were mixed with 300 µL of diluted carob molasses or fermented milk extract. Mixtures were shaken and kept in darkness at room temperature for 30 min before measuring absorbance at 765 nm using a spectrophotometer (Pg T80 + , England). TPC was expressed as gallic acid equivalent in mg/mL extract.

The antioxidant activity of fermented milk extracts and the diluted carob molasses were determined using DPPH and FRAP assays according to Lim and Quah (2007) and Oyaizu (1986), respectively.

For carrying out the DPPH assay, two milliliters of 0.15 mM DPPH dissolved in methanol were mixed with 1 mL of diluted carob molasses or fermented milk extracts, mixed well, and left in the dark for 30 min at room temperature. Absorbance (Abs) was measured at 517 nm against distilled water as a blank using a spectrophotometer (Pg T80 + , England). Control was prepared by adding 2 mL of DPPH to 1 mL of methanol. The radical scavenging activity of each extract was calculated using the following equation:

$${\text{Radical}}\,{\text{scavenging}}\,{\text{activity}}\,(\%) \, = \,(1\, - \,{\text{Abs}}\,\, {\text{sample}}\, / \, {\text{Abs}}\,\,{\text{control}}) \, \times \,100$$

For carrying out the FRAP assay, one milliliter of diluted carob molasses (1:100) or fermented milk extracts was added to 2.5 mL of phosphate buffer (0.1 M, pH 6.6) and 2.5 mL of potassium ferricyanide (1% w/v). The mixture was then incubated in a water bath at 50 °C for 20 min followed by cooling to room temperature and adding 2.5 mL of trichloroacetic acid (10% w/v). The contents of the tubes were centrifuged at 10,000 × g at 4 °C for 10 min. Next, 2.5 mL of supernatant was removed from each tube, and mixed with 2.5 mL of distilled water and 0.5 mL of ferric chloride solution (0.1% w/v). The mixtures were allowed to stand for 30 min then absorbance was measured at 700 nm using a UV/Visible spectrophotometer, Pharmacia-LKB-Ultrospec III (Pharmacia, USA). The FRAP values, expressed in mg gallic acid equivalents (GAE)/mL extract, were derived from a standard curve.

### Sensory evaluation

Fermented milk samples were evaluated for color, flavor, texture, and overall acceptability. The evaluation was conducted by 9 panelists who have experience in sensory evaluation of dairy products, from the Dairy Research Department, Food Technology Research Institute. Samples were presented in 100 mL-plastic cups for sensory evaluation at 1, 7, and 14 days of refrigerated storage. Water was provided between evaluations of samples for mouth rinsing. The evaluation was determined using a 9-point hedonic scale (1 dislike extremely, and 9 like extremely) according to El-Sayed et al. (2019).

### Statistical analysis

The data were analyzed by a general linear model procedure (GLM) using SAS statistical analysis software package (SAS Procedure Guide ‘Version 6.12 Ed.’ SAS Institute Inc., Cary, 2004). The statistical analysis was performed using a one-way analysis of variance (ANOVA). Means were compared by Duncan’s test at the significance level of p < 0.05.

## Results and discussion

### Carob molasses composition

Carob molasses has attracted consumers’ attention as an alternative natural sweetener, and for its content of phytochemicals, vitamins, and minerals (Goulas and Aresti, 2021). Also, carob molasses was characterized by a high reducing sugar content, functional properties, and high antioxidant activity (Tounsi et al., 2020). Table 1. Illustrates the chemical composition and antioxidant activity of carob molasses. Carob molasses contains a higher amount of TSS (~ 79%), total sugars (31.8%), and ash (2.15%), which referred to the high concentration degree of carob molasses by heat treatment. The current findings agree with those of Ibrahim et al. (2020). Also, the results showed that the diluted carob molasses (1:100 w/w) has a higher total phenolic content (3149 mg gallic acid equivalent/100 g), which is significantly higher than that determined in carob molasses (about 1400 mg GAE/100 g DM) in the previous studies (Tounsi et al., 2020). Phenolic compounds in carob belong mainly to the groups of phenolic acids, tannins, and flavonoids. They can be found in free, bound, or soluble conjugated forms, and they are an active source of antioxidants (Chait et al., 2020). The HPLC result shows that the individual phenolic compounds in carob molasses are gallic acid, chlorogenic acid, syringic acid, pyrocatechol, rutin, ellagic acid, coumaric acid, naringenin, taxifolin, cinnamic acid, and kaempferol (Table 1). It was observed that gallic acid was the predominant phenolic compound present in carob molasses (166.8 mg/100 g), followed by naringenin (5.31 mg/100 g) and then ellagic acid (3.029 mg/100 g). On the contrary, the compounds that are less present are syringic acid, pyrocatechol, and rutin (0.269, 0.234, and 0.254, respectively). The increase in the total phenolic content, and also the other constituents, is due to the evaporation process during heat treatments which increases the total solids content (Tounsi et al., 2020). TPC overwhelmingly appears strictly related to the antioxidant activity of carob molasses. The diluted carob molasses (1:100 w/w) showed a higher antioxidant activity which was measured by DPPH scavenging activity (75.23%), and FRAP (96.3 mg GAE/100 g). Also, it was observed that the carob molasses was higher in minerals such as Ca, K, Fe, Zn, and Mg.

**Table 1:** Chemical composition, phenolic profile, and antioxidant activity of carob molasses

Parameter	Carob molasses
TSS (%)	78.9 ± 0.65
Total sugars (%)	31.8 ± 0.46
Ash (%)	2.15 ± 0.052
Total phenolic content (mg GAE/100 g)*	3149 ± 0.27
DPPH scavenging activity (%)*	75.23 ± 1.02
FRAP (mg GAE/100 g)*	96.3 ± 0.041
Minerals (mg/100 g)	
Ca	167.00 ± 0.936
Fe	1.800 ± 0.110
K	666.30 ± 0.716
Zn	1.125 ± 0.115
Mg	1.883 ± 0.107
Individual polyphenols (mg/100 g)	
Gallic acid	166.848
Chlorogenic acid	1.50
Syringic acid	0.269
Pyro catechol	0.234
Rutin	0.254
Ellagic acid	3.029
Coumaric acid	0.665
Naringenin	5.310
Taxifolin	0.709
Cinnamic acid	1.124
Kaempferol	1.309

*GAE* Gallic acid equivalents

*These results for carob molasses diluted 1: 100 using water

### Physicochemical properties of bio-fermented goat milk

The main physicochemical characteristics (TS, fat, protein, and ash) of bio-fermented goat milk containing 0, 2, and 4% of carob molasses are presented in Table 2. The higher total solid content was recorded for CB4 treatment followed by CH4 treatment, while the lowest total solid content was found for CB1 and CH1 treatments. Thus, there is a linear correlation between the increased added amount of carob molasses and the total solid content of the bio-fermented milk samples. This observation could be mainly attributed to the higher content of total soluble solids in carob molasses. These results are in general agreement with those found by Kulcan et al. (2021). Also, Hassanein et al. (2014) noted that adding concentrated pomegranate extract to yogurt increased its total solids. Almosawi et al. (2015) reported that the total solids of yogurt supplemented with date syrup were higher than that of plain yogurt and the total solids significantly increased by increasing the concentration of date syrup. On the other hand, the current study showed that the addition of carob molasses had no significant (P < 0.05) effect on the protein and fat content of the fermented goat milk samples. The treatments CB1 and CH1 showed the lowest ash content, compared with carob molasses-containing treatments. The ash content in fermented goat milk was increased by adding carob molasses.

**Table 2:** Chemical composition (%) of fermented goat’s milk developed by using different levels of carob molasses and different strains of probiotic bacteria

	TS (%)	Fat (%)	Protein (%)	Ash (%)
CB	12.65 ± 0.0^D^	3.42 ± 0.06^A^	3.32 ± 0.04^A^	0.72 ± 0.02^B^
CB2	14.58 ± 0.26^C^	3.46 ± 0.07^A^	3.36 ± 0.17^A^	0.74 ± 0.02^AB^
CB4	17.23 ± 0.24^A^	3.44 ± 0.06^A^	3.36 ± 0.08^A^	0.75 ± 0.02^A^
CH	12.45 ± 0.11^D^	3.47 ± 0.04^A^	3.33 ± 0.07^A^	0.72 ± 0.01^B^
CH2	14.26 ± 0.2^C^	3.49 ± 0.04^A^	3.35 ± 0.20^A^	0.75 ± 0.01^A^
CH4	15.99 ± 0.1^B^	3.40 ± 0.09^A^	3.37 ± 0.09^A^	0.76 ± 0.01^A^

Values are means ± SD of three independent replicates. Means in the same column with different superscripts are significantly different (p < 0.05). TSS: total soluble solids. CB, CB2, CB4: fermented goat milk developed using *B. bifidum* and 0, 2, and 4% carob molasses. CH, CH2, and CH4: fermented goat milk developed using *L. helveticus* and 0, 2, and 4% carob molasses

### pH and viscosity of bio-fermented goat milk

Table 3 displays the changes that occurred in pH and viscosity values over the shelf life of the bio-fermented goat milk samples. The findings indicated that pH values gradually declined with the progress of cold storage time, thus the change in pH values occurred in a time-dependent manner. As well, pH changes occurred in a strain-dependent manner, displaying that samples containing *Bifidobacterium bifidum* DSM recorded the lowest pH values, compared to that containing *Lactobacillus helveticus* (CH5). The decline in pH values is associated with the metabolic activity of probiotic bacteria, which had the capability of degrading carbohydrates (lactose, and carob molasses carbohydrates) and producing organic acids. pH values continued to drop throughout the storage period, reaching between 4.18 to 4.0. Previous findings obtained by Chait et al. (2021) and Arab et al. (2022) showed a similar tendency associated with the pH decline, which resulted from adding carob powder to fermented milk and yoghurt.

**Table 3:** Changes in pH and viscosity values of fermented goat’s milk developed by using different levels of carob molasses and different strains of probiotic bacteria during cold storage

	pH			Viscosity (cP)		
	1 d	7 d	14 d	1 d	7 d	14 d
CB	4.47 ± 0.02^aC^	4.25 ± 0.02^bD^	4.13 ± 0.01^bC^	40.67 ± 0.46^aE^	35.17 ± 0.76^bE^	32.00 ± 1.00^cE^
CB2	4.41 ± 0.01^aD^	4.22 ± 0.01^bBC^	4.10 ± 0.01^cB^	58.23 ± 0.49^aC^	52.17 ± 0.76^bC^	45.00 ± 1.00^cC^
CB4	4.36 ± 0.02^aE^	4.16 ± 0.01^bC^	4.09 ± 0.02^cC^	78.33 ± 1.53^aA^	72.17 ± 1.26^bA^	64.00 ± 1.00^cA^
CH	4.67 ± 0.02^aB^	4.26 ± 0.03^bB^	4.18 ± 0.01^cB^	45.33 ± 1.53^aD^	39.00 ± 1.00^bD^	35.33 ± 0.58^cD^
CH2	4.60 ± 0.02^aB^	4.22 ± 0.02^bBC^	4.14 ± 0.02^bB^	64.67 ± 1.53^aB^	52.83 ± 0.76^bC^	48.67 ± 2.08^cB^
CH4	4.57 ± 0.01^aA^	4.20 ± 0.02^bA^	4.10 ± 0.03^cA^	76.33 ± 1.16^aA^	70.00 ± 1.00^bB^	64.33 ± 2.08^cA^

Values are means ± SD of three independent replicates. Means with different superscripts (A, B…) in each column at the same time for the various treatments are significantly different (P < 0.05) while means with different superscripts (a, b…) in each row at various storage times for the same treatment are significantly different (P < 0.05). cP: centipoise. CB, CB2, CB4: fermented goat milk developed using *B. bifidum* and 0, 2, and 4% carob molasses. CH, CH2, and CH4: fermented goat milk developed using *L. helveticus* and 0, 2, and 4% carob molasses

The carob molasses incorporation increased the viscosity of the bio-fermented goat milk during the storage period, compared to the unsupplemented samples. However, differences in viscosity were insignificant between carob molasses-containing samples and control samples. Moreover, the goat milk samples fermented with *Lactobacillus helveticus* CH5 were more viscous than those fermented with *Bifidobacterium bifidum* DSM, without any significant differences between them. This is probably explained by the ability of some probiotic strains to produce exopolysaccharides and thickening agents that can retain more water. The increased total solids resulting from adding carob molasses to the supplemented samples represent another factor in increasing viscosity. Viscosity significantly decreased (p < 0.05) with the progress of the storage period. Arab et al. (2022) reported almost similar findings and attributed this observation to the increased proteolytic activity and its impact on structure disintegration, modification, and disruption in the yoghurt protein network. For this reason, stabilizers and texture agents are used to improve the viscosity of fermented milk.

### Viable counts of probiotic strains

The viable bacterial counts of *Bifidobacterium bifidum* DSM and *Lactobacillus helveticus* CH5 in the resultant fermented goat’s milk are shown in Table 4. The current findings revealed that the viability of probiotic strains during cold storage was almost time-dependent and strain-dependent (p < 0.05). The viable counts of these two probiotic strains significantly (p < 0.05) increased with the progress of storage time and the increasing added amounts of carob molasses, ranging between 8.68 and 10.42 log CFU/g. Moreover, the two probiotic strains kept higher viable counts than the therapeutic levels (10^6^ cells per gram of mL of food) through the shelf life of fermented milk samples. *Bifidobacterium bifidum* DSM and *Lactobacillus helveticus* CH5 recorded higher viability percentages in fermented goat milk containing 4% carob molasses followed by that containing 2% carob molasses. Furthermore, the highest viability percentages were recorded at the final cold storage time. This higher viability at the final stage of storage time could be attributed to the carob molasses constituents, of which the total sugars represent the most abundant component. As well, the presence of dietary fiber (Carlson et al., 2018) and polyphenols (Owen et al., 2003) in carob extract and carob powder might participate in increasing the viable counts of probiotics. In a very recent study, Chait et al. (2021) exhibited that the viability and growth of *Lactobacillus brevis* have been improved by maintaining higher viable counts (8 log CFU/g) during the storage period by incorporating the carob powder. Carob dietary fiber is among the ingredients known for its prebiotic effect, which is highly involved in enhancing the viability of lactic acid bacteria. Moreover, carob polyphenol is another component known to improve the growth and viability of probiotics in milk. In this context, de Azevedo et al. (2018) displayed that the growth of *L. acidophilus* increased in fermented skim milk containing grape pomace extract rich in polyphenols compounds. Carob polyphenols also play an important role as hydrogen peroxide scavengers, thus protecting the probiotic strains during fermentation and storage (Chait et al., 2020). Generally, the incorporation of derivatives of food of plant origin can enhance the growth of lactic acid bacteria maintaining high viable numbers during storage due to its constituents including carbohydrates, fibers, and polyphenols which serve as a carbon source for probiotics. Furthermore, maintaining the viable counts of these probiotic strains in the current study could be partially explained by the absence of any competition with other lactic acid bacteria, *e.g.,* starter yoghurt culture. This suggests that carob molasses may be a significant component in extending the probiotic viability of fermented goat milk, which in turn positively affects human health.

**Table 4:** Viable counts of *Bifidobacterium bifidum* DSM and *Lactobacillus helveticus* CH5 in fermented goat’s milk developed by using different levels of carob molasses and different strains of probiotic bacteria during cold storage

Treatment	Probiotic strain	Viable counts (log CFU/mL)		
		1 d	7 d	14 d
CB	*Bifidobacterium bifidum*	8.68 ± 0.04^cE^	9.15 ± 0.02^bE^	9.82 ± 0.06^aD^
CB2		8.93 ± 0.02^cD^	9.66 ± 0.03^bC^	10.17 ± 0.06^aBC^
CB4		9.33 ± 0.04^cA^	9.82 ± 0.05^bA^	10.22 ± 0.04^aB^
CH	*L. helveticus*	8.75 ± 0.06^cE^	9.20 ± 0.01^bE^	10.08 ± 0.06^aC^
CH2		9.07 ± 0.07^cC^	9.33 ± 0.04^bD^	10.18 ± 0.04^aB^
CH4		9.18 ± 0.03^cB^	9.74 ± 0.04^bB^	10.42 ± 0.04^aA^

Values are means ± SD of three independent replicates. Means with different superscripts (A, B…) in each column at the same time for the various treatments are significantly different (P < 0.05) while means with different superscripts (a, b…) in each row at various storage time for the same treatment are significantly different (P < 0.05). CFU: colony forming unit. CB, CB2, CB4: fermented goat milk developed using *B. bifidum* and 0, 2, and 4% carob molasses. CH, CH2, and CH4: fermented goat milk developed using *L. helveticus* and 0, 2, and 4% carob molasses

### Phenolic content and antioxidant activity of bio-fermented goat milk

**Fig. 1:**
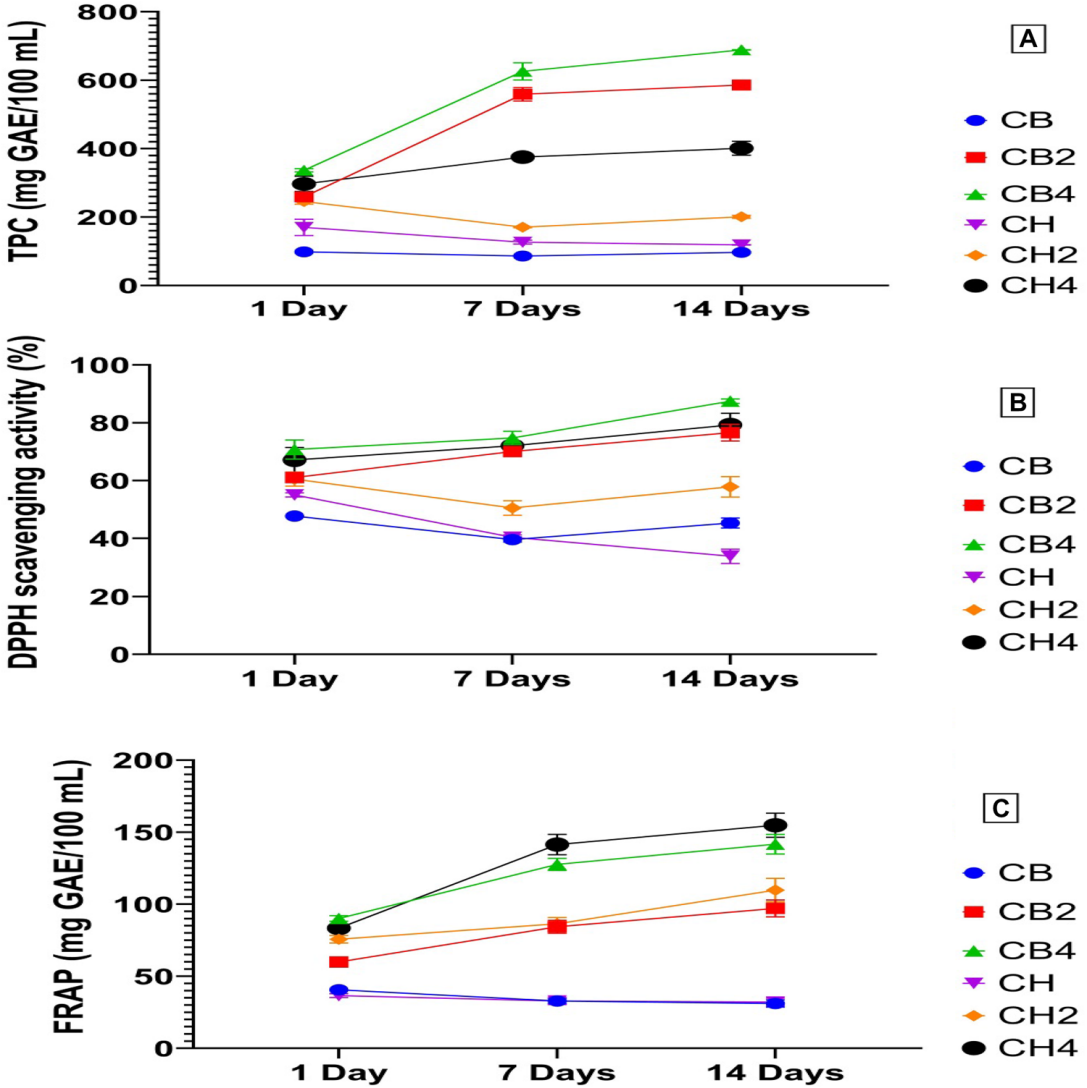
Changes in TPC (**A**), DPPH (**B**), and FRAP (**C**) values of fermented goat’s milk developed by using different levels of carob molasses and different strains of probiotic bacteria during cold storage. Values are means ± SD of three independent replicates. Means with different superscripts are significantly different (p < 0.05). *TPC* total phenolic content, *GAE* gallic acid equivalent, *DPPH* 2, 2-diphenyl-1-picrylhydrazyl, *FRAP* Ferric Reducing Antioxidant Power. CB, CB2, CB4: fermented goat milk developed using B. bifidum and 0, 2, and 4% carob molasses. CH, CH2, and CH4: fermented goat milk developed using L. helveticus and 0, 2, and 4% carob molasses

The changes in TPC of bio-fermented goat milk fortified with carob molasses were investigated during cold storage at 4 °C for 14 days (Fig. 1A). During the storage period, all treatments showed varying values of total phenolic content. Fermented goat milk (CB4) had the highest TPC throughout the entire storage period, while treatment CB had the lowest TPC content. The current findings are in agreement with Kulcan et al. (2021) who found that the TPC was increased in yoghurt by increasing the added amount of carob extract. Also, Dos Santos et al. (2017) found that the TPC in bio-fermented goat milk has been increased in samples supplemented with grape pomace extract. The differences among treatments in TPC may be due to the proteolytic activity of probiotic strains on milk proteins, which may release bioactive compounds during fermentation and storage such as amino acids with phenolic side chains (Abou-Soliman et al., 2022). Also, the increased addition of carob molasses increased the TPC in fermented milk. The content of phenolic compounds in CB1 and CH1 (98.26 and 169.78 mg GAE/100 g, respectively) is probably due to the presence of other compounds in milk other than polyphenols such as low molecular weight antioxidants, free amino acids, peptides, and proteins.

These findings confirmed the expected observation associated with increased antioxidant activity by increasing the total phenolic content (Fig. 1A). On the 1st day of cold storage, all treatments containing carob molasses showed an increase in DPPH radical scavenging activity (%), and the highest values were observed for treatments CH4 and CB4 which contained 4% carob molasses (Fig. 1B). On the 7th and 14th days of storage, the best DPPH scavenging activity was recorded for treatments CB4, CH4, and CB2. The treatment CB4 was the highest in DPPH scavenging activity during the entire storage period, compared to other treatments. The differences in the DPPH radical scavenging activity among goat-fermented milk could be attributed to the amount of carob molasses added to the fermented milk which affects the TPC. Also, the proteolysis degree by probiotic strains and the type of peptides released are considered other factors that participated in the observed increment in the antioxidant activity (Taha et al., 2017). The lowest DPPH scavenging activity was noted for CB and CH treatments during all storage times. The lowest DPPH scavenging activity was found with CB and CH treatments during all storage times. These results are similar to that found by Kulcan et al. (2021) who noted that the TPC and antioxidant activity values were found to be the lowest in yoghurt sample without carob extract (plain sample).

The results shown in Fig. 1C showed that FRAP values of fermented goat’s milk were influenced by the type of probiotic strain, and the added amount of carob molasses. The FRAP values have been increased with the increased added amount of carob molasses. On 1st day, treatment CB4 showed the highest FRAP value followed by CH4 treatment, while by the end of the storage period (14th day), the CB4 was the highest. These results are in general agreement with those found by Sah et al. (2015). The differences in FRAP values among all treatments during the storage period may also be attributed to the resultants formed by probiotic and starter cultures such as organic acids (Xiao et al., 2015). The observed increment in antioxidant activity as DPPH-scavenging activity and FRAP values could be attributed to the metabolic activity of probiotic strains and its capability in polyphenols bioconversion from polymeric polyphenols to monomeric ones.

### Sensory properties

Sensory properties are among the principal determinant factors of food quality. The descriptors evaluated in the current study, including color, flavor, texture, and total acceptance, are displayed in Table 5. The various descriptors that gained the highest scores were recorded for samples containing *B. bifidum* followed by that containing *L. helveticus* without adding carob molasses (CB and CH) on the first day of cold storage. However, almost insignificant changes were observed between all samples at the same corresponding storage time. Thus, adding 2, and 4% of carob molasses did not significantly change the scores acquired for all sensory descriptors. The progress of the cold storage period was the most effective factor in decreasing the sensory evaluation of the tested descriptors. Therefore, lower scores have been gained by all samples, in particular when adding 2, and 4% of the carob molasses, at the final storage period. The flavor was the most impacted descriptor and strongly decreased with the progress of storage time, in particular the unsupplemented samples (CB, and CH). These lower scores of flavor could be mainly attributed to the goaty flavor ascribed to goat milk products. On the other hand, samples containing 2, and 4% carob molasses had higher flavor scores resulting from the partial masking of this unpleasant flavor. Carob molasses contains a higher content of sugars which could positively impact some sensory descriptors including flavor, and color, and might influence the texture and hence, the overall acceptability. A similar tendency has been observed by Tami et al. (2022) who displayed that adding 8% sugar increased the sweetness of the product, covered the bitterness derived from plant polyphenols, and thus improved its acceptability.

**Table 5:** Organoleptic properties of fermented goat’s milk developed by using different levels of carob molasses and different strains of probiotic bacteria during cold storage

Storage time (day)	CB	CB2	CB4	CH	CH2	CH4
Color						
1 d	9.00 ± 0.50^aA^	8.33 ± 0.29^aA^	9.00 ± 0.00^aA^	9.00 ± 0.00^aA^	7.73 ± 0.50^aA^	8.83 ± 0.29^aA^
7 d	8.50 ± 0.50^aA^	7.83 ± 0.29^aAB^	7.67 ± 0.50^bA^	7.83 ± 0.29^bA^	7.67 ± 0.25^aAB^	7.83 ± 0.76^aAB^
14 d	7.50 ± 0.50^bA^	7.67 ± 0.58^aAB^	7.50 ± 0.58^bB^	7.57 ± 0.51^bAB^	7.50 ± 0.29^aAB^	7.33 ± 0.58^bAB^
Flavor						
1 d	6.33 ± 0.58^aA^	7.67 ± 0.58^aB^	8.83 ± 0.29^aA^	6.33 ± 0.58^aC^	7.33 ± 0.58^aA^	9.00 ± 0.00^aA^
7 d	5.83 ± 0.58^aAB^	7.17 ± 0.29^abA^	7.50 ± 0.50^bA^	5.33 ± 0.58^aB^	6.67 ± 1.15^aAB^	7.67 ± 0.58^bAB^
14 d	3.77 ± 0.40^bC^	6.33 ± 0.58^bB^	7.33 ± 0.58b^AB^	5.17 ± 1.04^aC^	6.33 ± 0.58^aB^	7.50 ± 0.50^bAB^
Texture						
1 d	7.33 ± 0.58^aA^	8.33 ± 0.29^aA^	8.50 ± 0.50^aA^	8.67 ± 0.58^aA^	8.50 ± 0.50^aA^	8.50 ± 0.50^aA^
7 d	6.50 ± 0.50^aAB^	8.0 ± 0.58^bB^	7.50 ± 0.50^bA^	8.17 ± 0.29^aA^	7.83 ± 0.50^abA^	7.33 ± 0.58^bAB^
14 d	6.50 ± 0.50^aAB^	6.33 ± 1.00^aC^	7.17 ± 0.29^bAB^	8.17 ± 0.29^aA^	7.33 ± 0.58^bAB^	7.25 ± 0.58^bAB^
Total acceptability						
1 d	9.0 ± 0.58^aA^	7.50 ± 0.50^aA^	8.67 ± 0.58^aA^	8.33 ± 0.58^aA^	7.50 ± 0.50^aA^	8.50 ± 0.50^aA^
7 d	7.33 ± 0.58^aA^	7.00 ± 0.00^aA^	7.83 ± 0.76^aA^	7.83 ± 0.29^aA^	7.83 ± 0.29^abA^	7.83 ± 0.29^abA^
14 d	5.33 ± 0.58^bA^	6.17 ± 0.29^bA^	7.50 ± 0.50^aA^	5.00 ± 0.00^bA^	7.50 ± 0.58^bA^	7.33 ± 0.29^bA^

Values are means ± SD of three independent replicates. Means with different superscripts (A, B…) in each column at the same time for the various treatments are significantly different (P < 0.05) while means with different superscripts (a, b…) in each row at various storage time for the same treatment are significantly different (P < 0.05). CB, CB2, CB4: fermented goat milk developed using *B. bifidum* and 0, 2, and 4% carob molasses. CH, CH2, and CH4: fermented goat milk developed using *L. helveticus* and 0, 2, and 4% carob molasses

In conclusion, this study proved that probiotic bacteria grow better when carob molasses is added, maintaining higher viable counts throughout the whole shelf life of the product. The carob molasses also boosts the cultures’ acidifying abilities and enhances the product’s viscosity. The findings also showed an increase in antioxidant activity, which is a reflection of the increased content of total phenolics. Carob molasses also had an impact on nearly all of the measured sensory descriptions, leading to partially covering up the unpleasant goaty flavor. Due to its stronger antioxidant activity and higher viable counts of probiotic bacteria, developed bio-fermented goat milk is considered a novel functional dairy beverage. Despite this, there is a need to reveal the health effects of fermented milk containing carob molasses. The effect of adding it, either before or after fermentation, can be studied to clarify the effect on bacterial viability and the bioconversion of phenolic compounds.

### Supplementary Information

Below is the link to the electronic supplementary material.

Supplementary file1 (DOCX 248 kb)
