# Supplementary figures and images for: Improving the physicochemical and antioxidative properties of fermented goat milk using carob molasses and some probiotic strains
Source: Food Sci Biotechnol. 2023 Aug 25;33(3):657–66. doi: 10.1007/s10068-023-01382-2 (PMC10805690; doi:10.1007/s10068-023-01382-2)

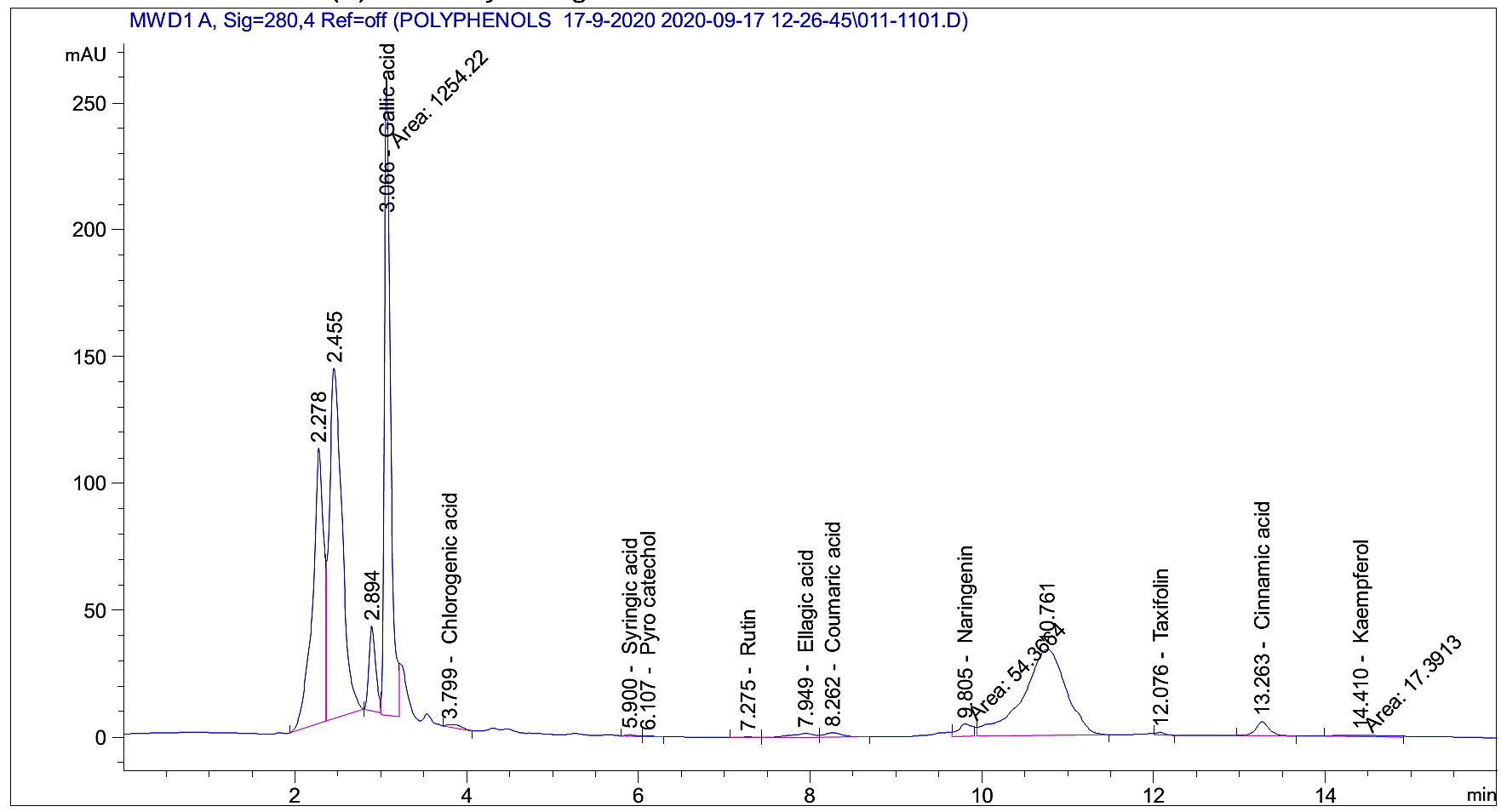

Supplement: Supplementary file 1 — Supplementary file1 (DOCX 248 kb) [file 10068_2023_1382_MOESM1_ESM.docx]
